# Time-restricted feeding improves metabolic flexibility, promotes beiging, and mitigates fibro-inflammation in the adipose tissue of aged mice

**DOI:** 10.1093/gerona/glag085

**Published:** 2026-03-30

**Authors:** Duraipandy Natarajan, Madison Milan, Zeke Reyff, Sharon Negri, Shoba Ekambaram, Rohan R Varshney, Michael C Rudolph, Stefano Tarantini, Priya Balasubramanian

**Affiliations:** Vascular Cognitive Impairment, Neurodegeneration, and Healthy Brain Aging Program, Department of Neurosurgery, University of Oklahoma Health Sciences Center, Oklahoma City, Oklahoma, United States; Oklahoma Center for Geroscience and Healthy Brain Aging, University of Oklahoma Health Sciences Center, Oklahoma City, Oklahoma, United States; The Peggy and Charles Stephenson Cancer Center, University of Oklahoma Health Sciences Center, Oklahoma City, Oklahoma, United States; Vascular Cognitive Impairment, Neurodegeneration, and Healthy Brain Aging Program, Department of Neurosurgery, University of Oklahoma Health Sciences Center, Oklahoma City, Oklahoma, United States; Oklahoma Center for Geroscience and Healthy Brain Aging, University of Oklahoma Health Sciences Center, Oklahoma City, Oklahoma, United States; The Peggy and Charles Stephenson Cancer Center, University of Oklahoma Health Sciences Center, Oklahoma City, Oklahoma, United States; Vascular Cognitive Impairment, Neurodegeneration, and Healthy Brain Aging Program, Department of Neurosurgery, University of Oklahoma Health Sciences Center, Oklahoma City, Oklahoma, United States; Oklahoma Center for Geroscience and Healthy Brain Aging, University of Oklahoma Health Sciences Center, Oklahoma City, Oklahoma, United States; The Peggy and Charles Stephenson Cancer Center, University of Oklahoma Health Sciences Center, Oklahoma City, Oklahoma, United States; Vascular Cognitive Impairment, Neurodegeneration, and Healthy Brain Aging Program, Department of Neurosurgery, University of Oklahoma Health Sciences Center, Oklahoma City, Oklahoma, United States; Oklahoma Center for Geroscience and Healthy Brain Aging, University of Oklahoma Health Sciences Center, Oklahoma City, Oklahoma, United States; The Peggy and Charles Stephenson Cancer Center, University of Oklahoma Health Sciences Center, Oklahoma City, Oklahoma, United States; Vascular Cognitive Impairment, Neurodegeneration, and Healthy Brain Aging Program, Department of Neurosurgery, University of Oklahoma Health Sciences Center, Oklahoma City, Oklahoma, United States; Oklahoma Center for Geroscience and Healthy Brain Aging, University of Oklahoma Health Sciences Center, Oklahoma City, Oklahoma, United States; Department of Biochemistry and Physiology and Harold Hamm Diabetes Center, University of Oklahoma Health Sciences Center, Oklahoma City, Oklahoma, United States; Department of Biochemistry and Physiology and Harold Hamm Diabetes Center, University of Oklahoma Health Sciences Center, Oklahoma City, Oklahoma, United States; Vascular Cognitive Impairment, Neurodegeneration, and Healthy Brain Aging Program, Department of Neurosurgery, University of Oklahoma Health Sciences Center, Oklahoma City, Oklahoma, United States; Oklahoma Center for Geroscience and Healthy Brain Aging, University of Oklahoma Health Sciences Center, Oklahoma City, Oklahoma, United States; The Peggy and Charles Stephenson Cancer Center, University of Oklahoma Health Sciences Center, Oklahoma City, Oklahoma, United States; Department of Health Promotion Sciences, College of Public Health, University of Oklahoma Health Sciences Center, Oklahoma City, Oklahoma, United States; Vascular Cognitive Impairment, Neurodegeneration, and Healthy Brain Aging Program, Department of Neurosurgery, University of Oklahoma Health Sciences Center, Oklahoma City, Oklahoma, United States; Oklahoma Center for Geroscience and Healthy Brain Aging, University of Oklahoma Health Sciences Center, Oklahoma City, Oklahoma, United States; The Peggy and Charles Stephenson Cancer Center, University of Oklahoma Health Sciences Center, Oklahoma City, Oklahoma, United States; Department of Biochemistry and Physiology and Harold Hamm Diabetes Center, University of Oklahoma Health Sciences Center, Oklahoma City, Oklahoma, United States

**Keywords:** Time-restricted feeding, Inflammation, Aging, Adipose tissue, Beiging

## Abstract

Adipose dysfunction contributes to age-related systemic decline primarily through its adverse effects on energy metabolism, insulin sensitivity, circulating adipokines, and inflammation. Time-restricted feeding (TRF) has emerged as a promising approach to correct adipose and metabolic dysfunction. However, most of these studies were carried out in young animals. Whether TRF could exert similar beneficial effects in the adipose tissue during aging remains unknown. To address this, 18-month-old C57BL/6 mice were placed on either a TRF diet (food intake restricted to a 6-h time window every day in the dark phase) or an unrestricted diet for 6 months. Young animals on an unrestricted diet acted as additional controls to compare the effects of aging. Here, we demonstrate that a 6-month TRF regimen induces a biphasic pattern in whole-body energy metabolism characterized by a selective increase in energy expenditure and oxygen consumption during the active dark phase, aligning with the feeding schedule. TRF increased uncoupling protein 1 (UCP1) expression in the white adipose tissue (WAT) and reverses age-associated whitening of brown adipose tissue (BAT) in aged mice. In addition, TRF selectively enhances mitochondrial metabolism in WAT depots. Furthermore, TRF reduces macrophage infiltration, induces a favorable shift in macrophage polarization (lower M1/M2 ratio), and decreases fibrosis in adipose tissue. Overall, our findings indicate that TRF promotes a metabolically beneficial adipose phenotype characterized by beiging and reduced fibro-inflammation during aging. These results underscore the potential of TRF as a dietary intervention to mitigate adipose dysfunction and promote metabolic health in the aging population.

## Introduction

Adipose tissue undergoes significant age-related changes in mass, distribution, composition, endocrine function, and metabolic flexibility. Tissue-level changes, including increased adiposity, a shift in distribution from subcutaneous to visceral depots, and a decline in brown adipose tissue (BAT) mass and thermogenic function, begin to manifest around middle-age.^1^^,^^2^ At the cellular level, age-related impairment in adipogenesis and angiogenesis results in hypertrophy of existing adipocytes, triggering a cascade of events such as hypoxia and macrophage infiltration, leading to a chronic state of low-grade inflammation.^3^ Moreover, unresolved hypoxia and inflammation promote unfavorable remodeling and excessive production of extracellular matrix (ECM) proteins like collagen, resulting in fibrosis.^4^ Age-associated increases in senescence in stromal vascular cells, particularly adipose progenitor cells^5^ and macrophages,^6^^,^^7^ further exacerbate tissue dysfunction and perpetuate the inflammatory milieu. Together, these fibro-inflammatory pathological changes in the adipose tissue contribute to alterations in adipokine secretion, ectopic lipid deposition, and systemic insulin resistance, thereby increasing the risk for several age-related diseases,^3^ potentially also including vascular cognitive impairment and dementia (VCID).^8–10^ Such low-grade systemic inflammation, measurable through clinical markers like the neutrophil-to-lymphocyte ratio (NLR), has been shown to predict multimorbidity and mortality in older adults,^8^ further underscoring the systemic impact of adipose tissue immune dysregulation. Notably, single-cell sequencing studies that comprehensively mapped tissue-specific changes across the mouse lifespan reveal a striking finding that white adipose tissue (WAT) exhibits the earliest and the most pronounced age-related inflammatory changes.^9^^,^^10^ Collectively, these findings highlight adipose tissue as a key driver of systemic aging and a potential modulator of age-related disease risk.

Several dietary interventions known to enhance healthspan and lifespan have also been shown to favorably alter adipose tissue function.^11–13^ In particular, calorie restriction (CR) leads to significant adaptations in adipose tissue, including reduced mass, improved redox metabolism, increased adiponectin secretion, enhanced adipogenesis, and decreased senescence and inflammation.^11^^,^^14^^,^^15^ While the benefits of CR have traditionally been attributed to reduced caloric intake, recent studies suggest that inadvertent fasting imposed in CR studies significantly contributes to the geroprotective effects of CR.^16^ Consistent with this idea, time-restricted feeding (TRF) has emerged as an alternative dietary strategy to CR, wherein the food intake is restricted daily to a limited window of time without reducing the overall calorie intake.^17^ As observed with CR, several preclinical and clinical studies have reported similar beneficial outcomes with TRF regimens on glucose metabolism,^18^^,^^19^ hepatosteatosis,^20^ blood pressure,^21^ and lipid profile.^21^ Additionally, TRF enhances adipogenesis and improves the immune landscape and inflammation in adipose tissue.^22^^,^^23^ Importantly, intermittent fasting strategies have been reported to activate nonshivering thermogenesis (or increased cellular energy expenditure) in WAT, contributing to their metabolic benefits.^24^^,^^25^ This process is primarily driven by the emergence of brown-like adipocytes within WAT, commonly referred to as beige adipocytes. These cells mediate thermogenesis in WAT through the upregulation of uncoupling protein-1 (UCP1), a key thermogenic protein that uncouples mitochondrial electron transport chain activity from ATP synthesis, thereby generating heat. However, most studies demonstrating these effects have been conducted in young, diet-induced obese mouse models or overweight/obese human subjects. Whether TRF elicits similar benefits in adipose tissue during aging remains largely unknown.

To address this gap, we conducted a cross-sectional study investigating the effects of TRF (6 h daily during the active cycle) in late-middle-aged C57BL/6J mice (18-month old). We evaluated the impact of TRF on adipose tissue beiging, mitochondrial function, inflammation, and fibrosis. Our findings demonstrate that TRF improves metabolic flexibility, couples energy expenditure with energy availability, upregulates UCP1 expression and restores brown adipose tissue (BAT) phenotype, enhances mitochondrial function, and ameliorates age-associated fibro-inflammation in adipose tissue. These results suggest that TRF may represent a promising nonpharmacological strategy to reverse age-related adipose dysfunction.

## Methods

### Animals and treatment

Young (6-month old) and aged (18-month old) male C57BL/6J mice were purchased from Jackson Laboratories. Following an acclimatization period, animals were divided into three groups: (1) young mice fed ad libitum for 6 months, (2) aged mice fed ad libitum for 6 months starting at 18 months of age, and (3) aged mice subjected to a TRF regimen for 6 months starting at 18 months of age. All mice were housed under reverse light cycle conditions in a conventional animal facility with a 12:12-h light-dark cycle and unlimited access to water. The TRF regimen restricted food access to a 6-h window (10:00 am to 4:00 pm, dark phase in the reverse light cycle room), followed by an 18-h fasting period each day. Body composition was measured at baseline and 6-month post initiation of TRF using an EchoMRI Body Composition Analyzer (Houston, TX, USA). All animal procedures were approved by the Institutional Animal Care and Use Committee of the University of Oklahoma Health Sciences Center.

### Indirect calorimetry

During the final month of the TRF regimen, mice were transferred to indirect calorimetry cages (Promethion CAB16 system, Sable systems, NV) within the environmental cabinet maintained at 20 °C and the reversed day/night cycle was preserved. Animals were individually housed and maintained on their respective dietary regimens (ad libitum vs TRF) throughout the duration of the metabolic phenotyping measurements. Following a 48-h acclimation period, body weight (BW), energy expenditure (EE), and oxygen consumption (VO_2_) were continuously recorded for 96 h. Respiratory exchange ratio (RER), carbohydrate, and fat oxidation were calculated using the following equations:


$$\mathrm{RER}=VCO_{2}/VO_{2},$$

$$Carbohydrate\;\mathrm{oxidation}\;\left(g/min\right)=4.56\times VCO_{2}-3.21\times VO_{2},$$

$$\mathrm{Fat}\;\mathrm{oxidation}\;\left(g/\min\right)=1.69\times VO_{2}-1.70\times VCO_{2}.$$

### *In situ* mitochondrial ETC activity analysis

Mitochondrial ETC activity in freshly isolated adipose tissues was assessed by staining with the redox dye 2,3,5-triphenyltetrazolium chloride (TTC) as described previously.^26^ Adipose tissue (50-75 mg) was roughly minced into small pieces and incubated in 1% TTC prepared using phosphate-buffered saline at 37 °C for 30 min. Insoluble red formazan, which is the reduced form of TTC, was extracted by overnight incubation in 100% isopropanol at room temperature (RT). The absorbance of isopropanol was measured at 485 nm using a plate reader, and the OD was normalized to the tissue weight.

### Western blotting

Adipose tissue samples were homogenized in ice-cold RIPA buffer (Millipore Sigma, #R0278) supplemented with Halt protease and phosphatase inhibitor cocktail (Thermo Scientific, #PI78440). The lysates were placed in a rotating chamber in a cold room for 1 h and centrifuged at 16 000 *g* for 10 min. The clear supernatant beneath the lipid layer was separated and centrifuged again until all lipid traces were eliminated from the lysate. Protein concentrations in the isolated lysate were assessed using the Pierce BCA Protein Assay Kit (Thermo Scientific, #23227). Equal amounts of protein (30 µg) were separated on a 4%-20% SDS gel under reduced and denatured conditions, followed by wet transfer to a PVDF membrane (Bio-Rad). The transfer was confirmed using Ponceau staining and imaging, followed by washing twice with Tris-buffered saline with 1% tween 20 (TBST) for 5 min each. After blocking for 1 h with 5% nonfat milk at RT, the membranes were incubated overnight in a cold room with anti-UCP1 (1:1000, R&D Systems #MAB6158). Following washing with TBST thrice, the membranes were incubated with an HRP-conjugated anti-mouse secondary antibody (1:10000, Abcam #ab205719) for 1 h at RT. Following 3x TBST washes, the bands were developed using Pierce SuperSignal West Pico Plus chemiluminescent substrate, and the signals were detected using the ChemDoc imaging system. Densitometry was performed using Fiji ImageJ software, and the values were normalized to Ponceau staining.

### Histology and immunohistochemistry

Formalin-fixed and paraffin-embedded adipose tissues were sectioned at 5 µm thickness and mounted on positively charged slides. Slides were transferred to the Leica Bond RX for dewaxing and then treated for target retrieval at 100 °C for 20 min in a retrieval solution at pH 6.0. The sections were incubated with 5% goat serum (ThermoFisher Scientific #01-6201) for 30 min. Endogenous peroxidase was blocked using a peroxidase-blocking reagent, followed by incubation with the following primary antibodies: UCP1 (Abcam #ab10983), F4/80 (Abcam #ab111101), CD163 (1:600, Abcam #ab182422), and CD80 (1:500, Abcam #ab134120) for 60 min. For the secondary antibody, Poly-HRP IgG reagents were used. Detection was done using 3, 3′-diaminobenzidine tetrahydrochloride (DAB) as chromogen and counterstained with hematoxylin. Completed slides were dehydrated (Leica ST5020) and mounted (Leica MM24). Digital images were acquired using the Zeiss Axioscan Microscopic slide scanner. At least five representative images were obtained from each tissue (3 tissue sections per slide) for each depot per animal at 10x magnification. The number of positive cells was counted using the Fiji ImageJ software. Adipocyte area measurements were performed in H&E-stained slides using the AdipoSoft plug-in for the ImageJ software. The skeletonized outline of the adipocytes was double-checked for accuracy before quantification.

### Trichrome staining

Fibrosis was assessed using the Trichrome staining kit (Abcam; ab150686) according to the manufacturer’s instructions. Briefly, 15 µm thickness of formalin-fixed adipose tissue sections were deparaffinized with xylene and hydrated with distilled water. The slides were then placed in the preheated Bouin’s Fluid for 60 min, followed by cooling for 10-min. After rinsing with distilled water, the slides were stained with working Weigert’s Iron Hematoxylin for 5 min. Subsequently, slides were rinsed with distilled water for 2 min and counterstained with Biebrich Scarlet/Acid Fuchsin Solution. After 15 min, the tissue sections were differentiated in Phosphomolybdic/Phosphotungstic Acid Solution, followed by incubation in aniline blue Solution for 10 min. The slides were then thoroughly rinsed with distilled water and placed in 1% Acetic Acid Solution for 5 min. Finally, all the slides were dehydrated with ethanol and cleared with xylene solution and mounted in synthetic resin (DPX Mountant for Microscopy; 13510). The images were acquired using a ZEISS Axioscan 7 Microscope, and blue-stained areas were quantified using Fiji ImageJ software.

### Sirius Red/Fast Green collagen staining

The semiquantitative collagen detection kit for total tissue (Sirius Red/Fast Green collagen staining kit, Chondrex, Inc., catalog # 9046, Woodinville, WA) was used to assess the total amount of collagen and noncollagenous proteins in adipose tissue sections from the three experimental groups. Sections were stained with two dyes, Sirius Red and Fast Green. Sirius Red stains all types of collagens; conversely, Fast Green binds to noncollagenous proteins. To measure these parameters, 15 µM paraffin adipose tissue sections were deparaffinized by immersing the sections in xylene for 10 min. The sections were then transferred to a solution containing 1-part xylene and 1 part 100% ethanol for 10 min. The sections were then moved to 100% ethanol for 10 min. Tissue sections were then moved to a 50% ethanol solution in ddH_2_O for 5 min. Slides containing the tissues were then rinsed with ddH_2_O for 5 min. After this, slides were transferred to petri dishes and stained with the Sirius Red Fast Green dye solution for 30 min. The dye solution was then aspirated from the tissue sections, and the sections were rinsed with ddH_2_O until the water eluted from the sections ran clear. Stained tissues then had the dye eluted from them by adding the dye extraction buffer to each sample and gently mixing by pipetting until all the color was removed from the tissue sections. Eluted solutions were collected to measure the micrograms of collagen and noncollagenous proteins in each tissue section (3 per animal and adipose depot) reader at OD 540 (Sirius Red) and OD 605 (Fast Green) using a Tecan SPARK^®^ Multimode Microplate reader. Results were quantified and normalized to transform results as % collagen per protein by dividing the obtained collagen value in micrograms by the obtained value of noncollagenous proteins in micrograms to give an accurate representation of the levels of collagen relative to the amount of total protein in each tissue section.

### Real-time PCR

Total RNA was extracted from WAT and BAT depots with Trizol using the DirectZol RNA Microprep Kit (Zymo Research). Equal amounts of RNA (500 ng) were reverse-transcribed to cDNA using the High-Capacity RNA-to-cDNA kit (Thermo Scientific #4388950). Quantitative real-time PCR analysis was performed with equal quantities of cDNA using QuantStudio 12 Flex (Applied Biosystems). The Ct values were normalized to the housekeeping gene, GAPDH, and the relative mRNA concentrations of our target genes were assessed by the ΔΔct method. The primer sequences are provided in Table 1.

**Table 1:** Primer sequences (mouse) used for real-time PCR analysis.

Mouse primer name	Sequence
MCP1-F	GCAGTTAACGCCCCACTCA
MCP1-R	TCCAGCCTACTCATTGGGATCA
IL-6 F	ACAAGTCGGAGGCTTAATTACACAT
IL-6 R	TTGCCATTGCACAACTCTTTTC
IL-1b F	CACAGCAGCACATCAACAAG
IL-1b R	GTGCTCATGTCCTCATCCTG
TNFa-F	ATGAGAAGTTCCCAAATGGC
TNFa-R	CTCCACTTGGTGGTTTGCTA
GAPDH-F	AAGGTCATCCCAGAGCTGAA
GAPDH-R	CTGCTTCACCACCTTCTTGA
Beta-actin F	TGTCCACCTTCCAGCAGATGT
Beta-actin R	AGCTCAGTAACAGTCCGCCTAG
F4/80-F	TGCATCTAGCAATGGACAGC
F4/80-R	GCCTTCTGGATCCATTTGAA
CD163-F	CATGTCTCTGAGGCTGACCA
CD163-R	TGCACACGATCTACCCACAT
CD80-F	CCATGTCCAAGGCTCATTCT
CD80-R	TTCCCAGCAATGACAGACAG
Arg1-F	CAGAACCTGCTGTCCTGTGA
Arg1-R	TGTCGTTGGAATCAACCTGA
iNOS-F	CACCTTGGAGTTCACCCAGT
iNOS-R	ACCACTCGTACTTGGGATGC
UCP1-F	GTGAACCCGACAACTTCCGAA
UCP1-R	TGAAACTCCGGCTGAGAAGAT
Cidea-F	CTAGCACCAAAGGCTGGTTC
Cidea-R	CACGCAGTTCCCACACACTC
Dio2-F	CCAGCACCGGAAAGAGGAAA
Dio2-R	TCCTTGCACCATGACCCAAA
Prdm16-F	CAGCACGGTGAAGCCATTC
Prdm16-R	GCGTGCATCCGCTTGTG
Cox8b-F	TGCGAAGTTCACAGTGGTTC
Cox8b-R	TCAGGGATGTGCAACTTCA

Abbreviations: GADPH, Glyceraldehyde-3-phosphate dehydrogenase; IL, Interleukin; PCR, Polymerase chain reaction; TFN, Tumor necrosis factor.

### Statistical analysis

Statistical analyses were performed using GraphPad Prism 9.3.1 (GraphPad Software, San Diego, CA, USA), and the data are expressed as mean ± SEM. Data were analyzed by one-way ANOVA and Tukey’s multiple comparisons test. *p* < .05 was considered statistically significant.

## Results

### TRF induces a biphasic diurnal pattern in whole-body energy metabolism

**Figure 1:**
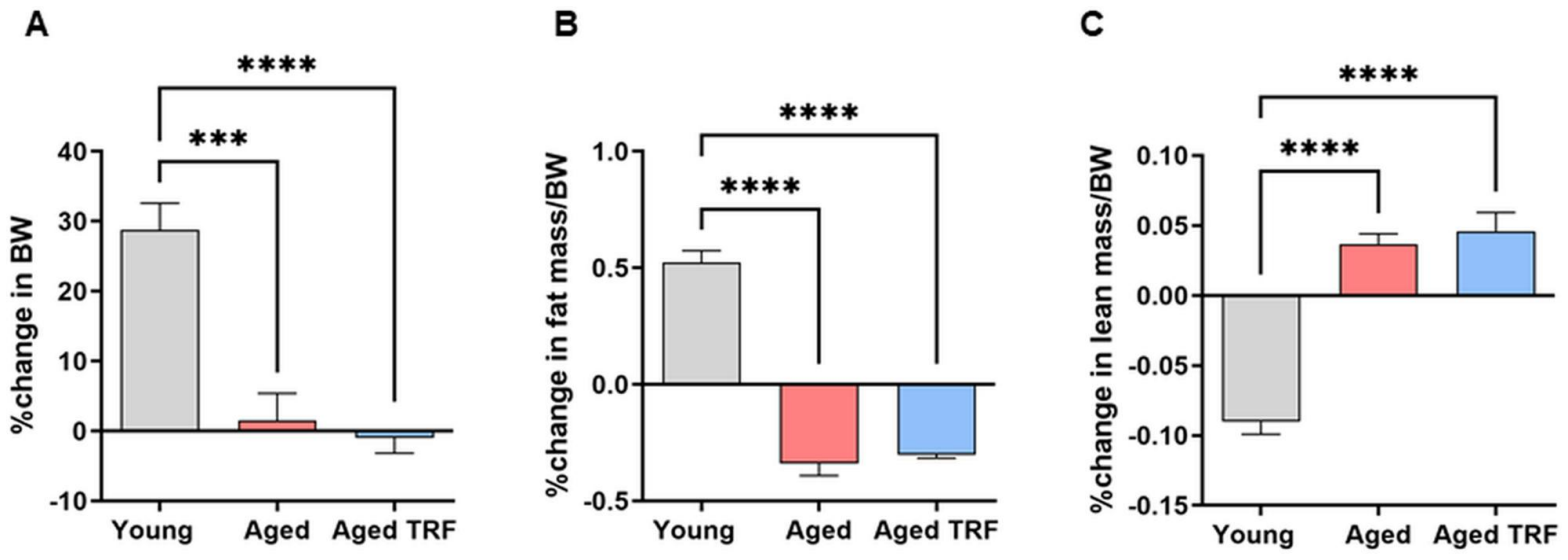
Effects of TRF on final body weight and composition. (A) Percent change in BW. (B, C) Percent change in fat mass and lean mass, respectively, as a function of BW. *n* = 6/group, ***p < .005 and ****p < .001 between the indicated groups. BW, body weight; TRF, time-restricted feeding.

**Figure 2:**
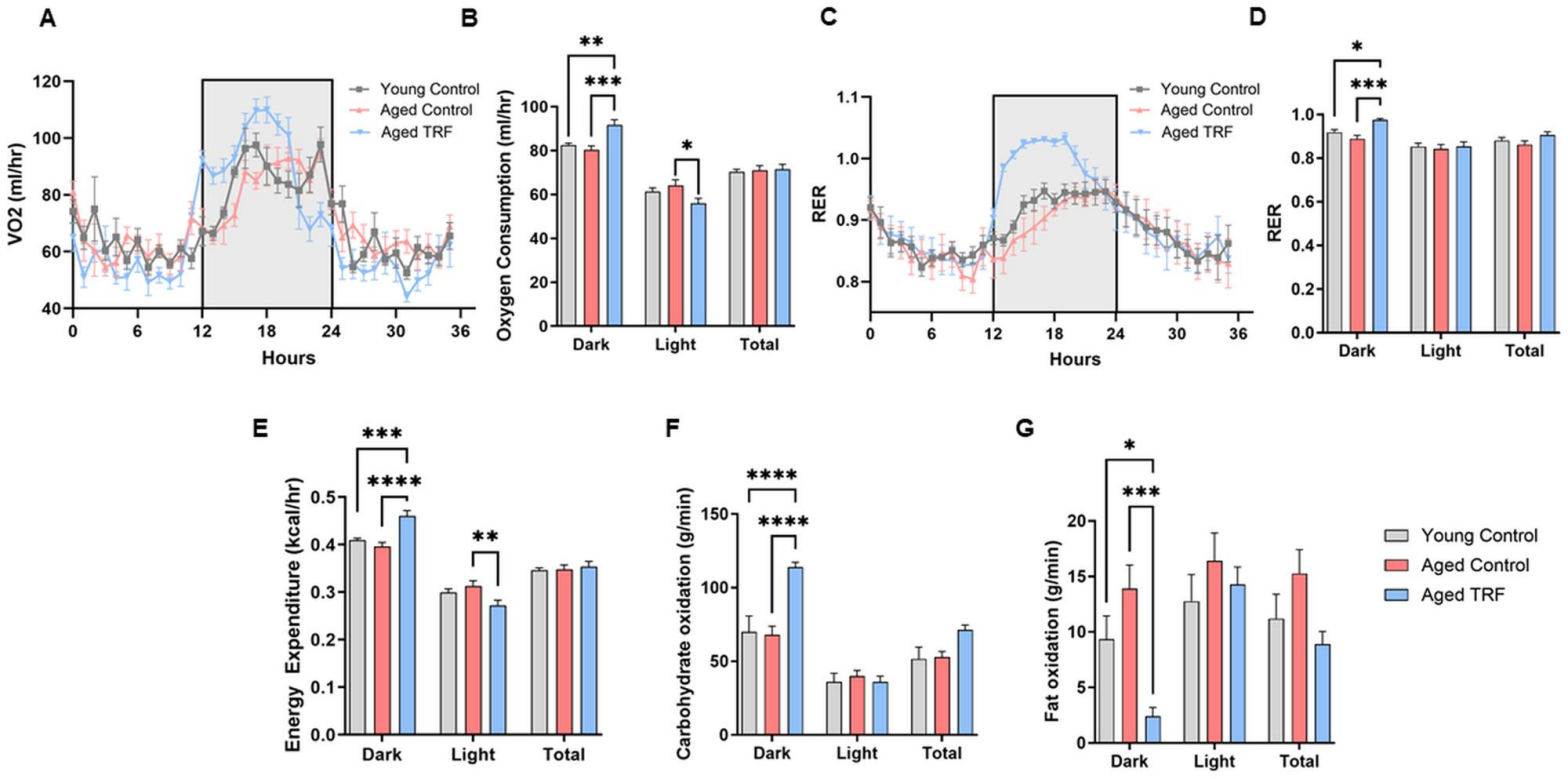
TRF Promotes metabolic adaptation in aged mice. (A, B) Oxygen consumption (mL/h). (C, D) RER. (E) Energy expenditure (kcal/h). (F) Carbohydrate oxidation (g/min) and (G) fat oxidation (g/min) measured during the dark, light, and over the total 24 h period. The grey shaded area represents the dark phase. *n* = 6-7/group, *p < .05, **p < .01, ***p < .005, and ****p < .001 between the indicated groups. RER, respiratory exchange ratio; TRF, time-restricted feeding; VO2, oxygen consumption.

To assess the impact of TRF on energy and adipose tissue metabolism in aging, we subjected 18-month-old male mice to 6 months of TRF, during which food access was limited to a 6-h window in the dark cycle each day. Young animals progressively gained body weight and fat mass during the experimental period (Supplementary Figure S1). In contrast, aged animals, both ad libitum–fed and TRF-fed, maintained their body weight and fat mass throughout the intervention (Supplementary Figure S1). However, at the study endpoint, final body weight and fat mass were significantly lower in aged mice, irrespective of diet, with a concurrent increase in lean mass compared to young controls (Figure 1A-C). Consistent with our previous study,^27^ TRF did not affect total food intake in this study. To further investigate the effects of TRF on whole-body energy metabolism during aging, we performed indirect calorimetry measurements in experimental mice. Aged mice subjected to TRF displayed a distinct biphasic diurnal pattern in oxygen consumption. TRF significantly increased oxygen consumption during the dark phase, specifically during the first 6 h (12 to 18 h on the timeline), which is the feeding period for this group. In contrast, TRF decreased oxygen consumption during the light phase (fasting period; Figure 2A and B). TRF also elicited a similar pattern with energy expenditure in aged mice (Figure 2E). During the dark phase, aged TRF mice also exhibited a significantly higher RER (close to 1) compared to aged and young ad libitum controls (Figure 2C and D). Consistent with this, carbohydrate oxidation rates were significantly elevated, while fat oxidation rates were reduced, in aged TRF mice relative to controls during the dark phase (Figure 2F and G). These diurnal shifts in energy metabolism parallel the imposed feeding schedule, indicating that TRF promotes metabolic adaptation to energy availability in aged mice.

### TRF promotes beiging of WAT in aged mice

**Figure 3:**
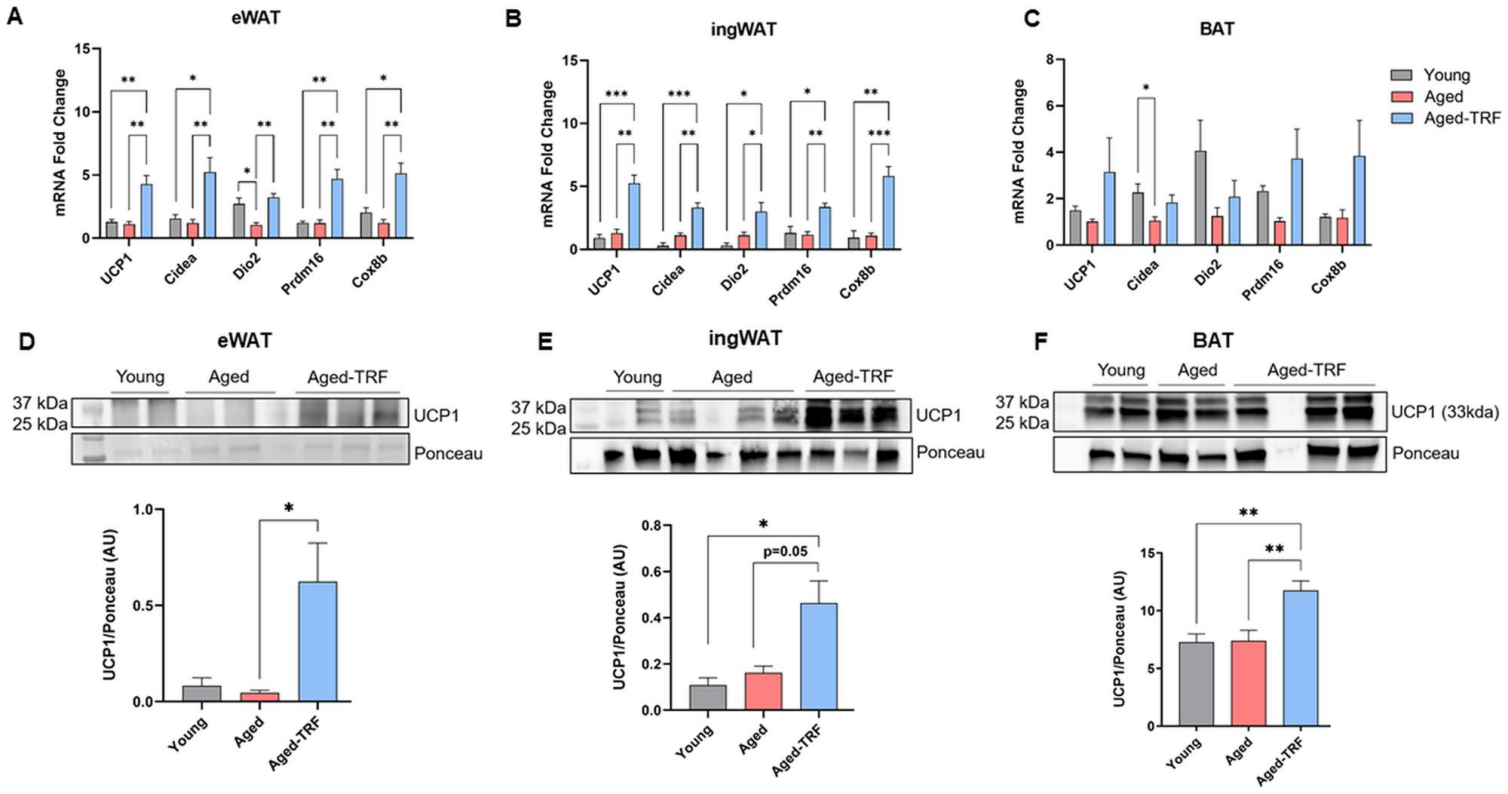
TRF Increases the expression of thermogenic genes in WAT of aged mice. (A-C) Real-time PCR analysis of the expression of thermogenic genes in eWAT, ingWAT, and BAT, respectively, *n* = 5-6/group. (D-F) Representative images of western blots and densitometry analysis for UCP1 protein levels after total protein normalization using ponceau staining in eWAT, ingWAT, and BAT, respectively, *n* = 5-6/group. Data are presented as mean ± SEM, *p < .05, **p < .01, and ***p < .005 between the indicated groups. AU, arbitrary units; BAT, brown adipose tissue; eWAT, epididymal white adipose tissue; ingWAT, inguinal white adipose tissue; PCR, polymerase chain reaction; TRF, time-restricted feeding; UCP1, uncoupling protein 1; WAT, white adipose tissue.

Intermittent fasting enhances adipose nonshivering thermogenesis by increasing UCP1 expression and promoting the beiging of WAT in young animals.^24^^,^^25^ Hence, we investigated whether TRF elicits a similar thermogenic response in aged mice. Gene expression analysis revealed that TRF upregulated a broad panel of thermogenic genes, including *Ucp1*, *Cidea*, *Dio2*, *Prdm16,* and *Cox8b* in both epididymal WAT (eWAT) and inguinal WAT (ingWAT) of aged mice (Figure 3A and B). Although a similar trend for an increase in the expression of thermogenic genes was observed in the BAT, it did not reach statistical significance due to high variability (Figure 3C).

**Figure 4:**
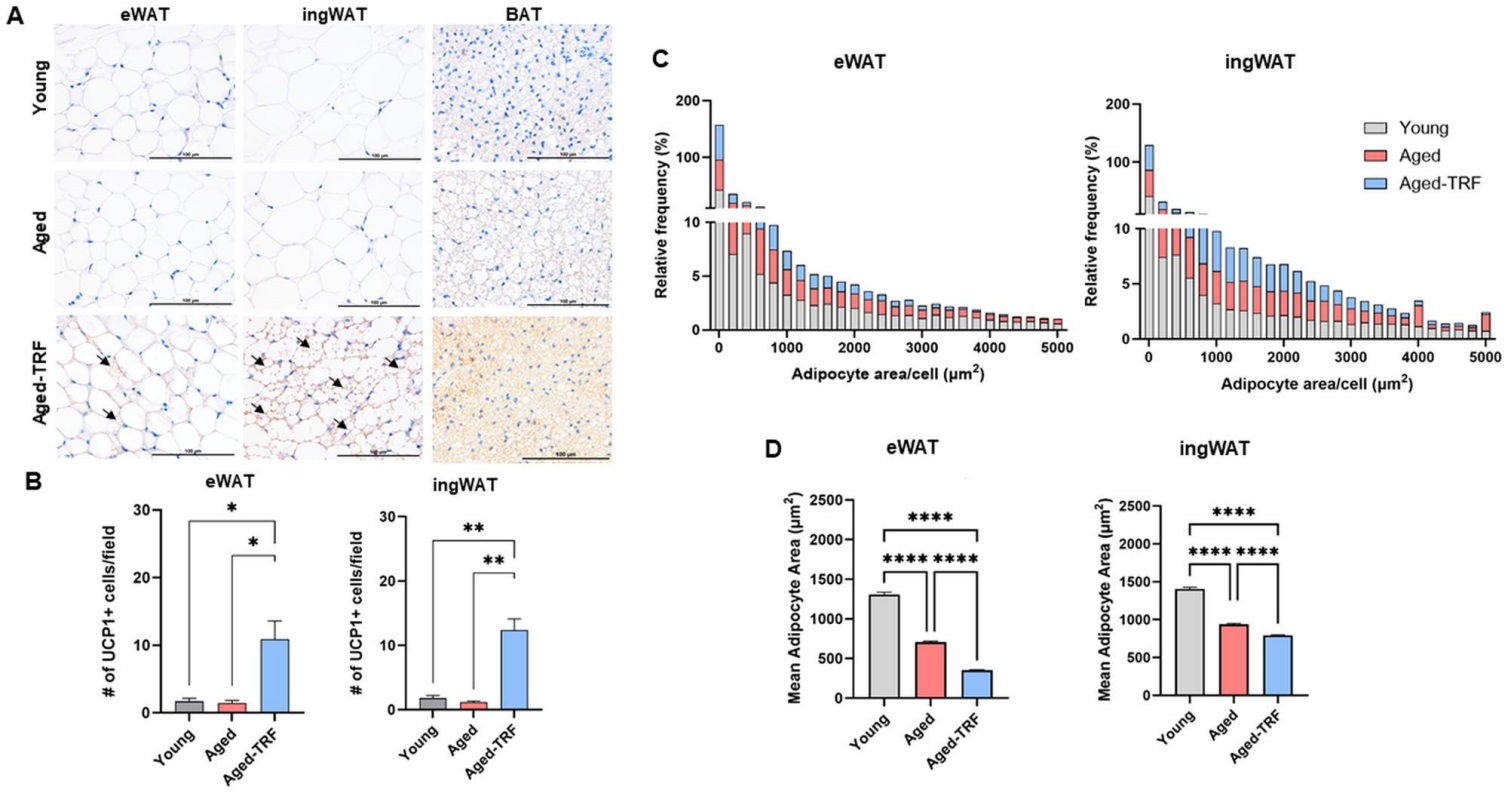
TRF Promotes beiging in WAT and reverses age-induced whitening of BAT. (A) Representative images of UCP1 immunohistochemistry on sections of eWAT, ingWAT, and BAT from young, aged, and aged-TRF mice. Black arrows point to multilocular UCP1+ beige adipocytes in WAT depots. (B) Quantification of the number of UCP1+ adipocytes in eWAT and ingWAT. The values represent the average number of UCP1+ adipocytes counted in at least five independent fields of the adipose tissue sections. (C, D) Adipocyte size distribution and the mean adipocyte area (µm^2^) in eWAT and ingWAT, respectively. Data are presented as mean ± SEM, *n* = 3-6/group. *p < .05, **p < .01, ***p < .005, and ****p < .001 between the indicated groups. BAT, brown adipose tissue; eWAT, epididymal white adipose tissue; ingWAT, inguinal white adipose tissue; TRF, time-restricted feeding; UCP1, uncoupling protein 1; UCP1+, uncoupling protein 1 positive; WAT, white adipose tissue.

To further evaluate whether TRF-induced thermogenesis in aged adipose tissue was associated with beiging, we performed western blotting and histological analyses. Consistent with gene expression data, UCP1 protein levels were elevated in both WAT depots (Figure 3D and E). TRF similarly increased UCP1 protein levels also in BAT of aged mice (Figure 3F). Histological examination revealed a significant increase in UCP1-positive multilocular adipocytes, which is a classical hallmark of beige adipocytes, in both eWAT and ingWAT (Figure 4A and B). While aging was associated with whitening of BAT, characterized by enlarged lipid droplets resembling white adipocytes, TRF restored the classical multilocular morphology and UCP1 expression in aged BAT (Figure 4A). Additionally, TRF animals exhibited markedly smaller adipocytes in both WAT depots compared to age-matched ad libitum-fed and young controls (Figure 4C and D). Interestingly, aged mice also showed smaller average adipocyte area in WAT relative to young controls (Figure 4D). These findings indicate that TRF promotes beiging of WAT through the induction of UCP1+ multilocular adipocytes and also reverses age-associated whitening of BAT.

### TRF increases mitochondrial respiration in the adipose tissue of aged mice

**Figure 5:**
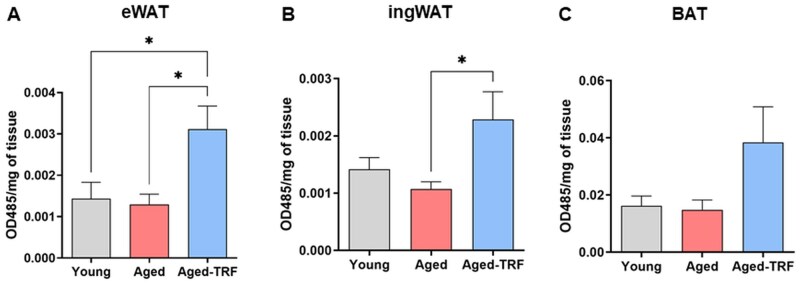
TRF increases mitochondrial ETC activity in WAT of aged mice. Mitochondrial ETC activity was assessed by measuring the reduction of the electron acceptor dye TTC in eWAT, ingWAT, and BAT depots (*n* = 4-7/group). Data are shown as mean ± SEM, *p < .05 between the indicated groups. BAT, brown adipose tissue; ETC, electron transport chain; eWAT, epididymal white adipose tissue; ingWAT, inguinal white adipose tissue; TRF, time-restricted feeding; TTC, 2,3,5-triphenyltetrazolium chloride; WAT, white adipose tissue.

One of the major characteristics of beige adipocytes is increased mitochondrial biogenesis and function. Therefore, we next sought to determine whether TRF enhances mitochondrial respiration in the adipose tissue of aged mice. To this end, we assessed mitochondrial electron transport chain (ETC) activity *in situ* in freshly isolated adipose tissue using the redox-sensitive dye TTC. TTC is reduced by mitochondrial ETC enzymes to an insoluble red formazan compound. The intensity of the red coloration is directly proportional to mitochondrial ETC activity and serves as a surrogate marker for mitochondrial respiration in live tissues. TRF significantly increased mitochondrial ETC activity in both eWAT and ingWAT when compared to their age-matched controls (Figure 5A and B). Validating the technique, the basal mitochondrial respiration in BAT was, as expected, at least ∼10-fold higher than in WAT. Although aged-TRF animals exhibited a trend toward increased BAT mitochondrial respiration, the difference did not attain statistical significance (Figure 5C).

### TRF reduces macrophage infiltration, drives M2 polarization, and attenuates inflammation in the aged adipose tissue

**Figure 6:**
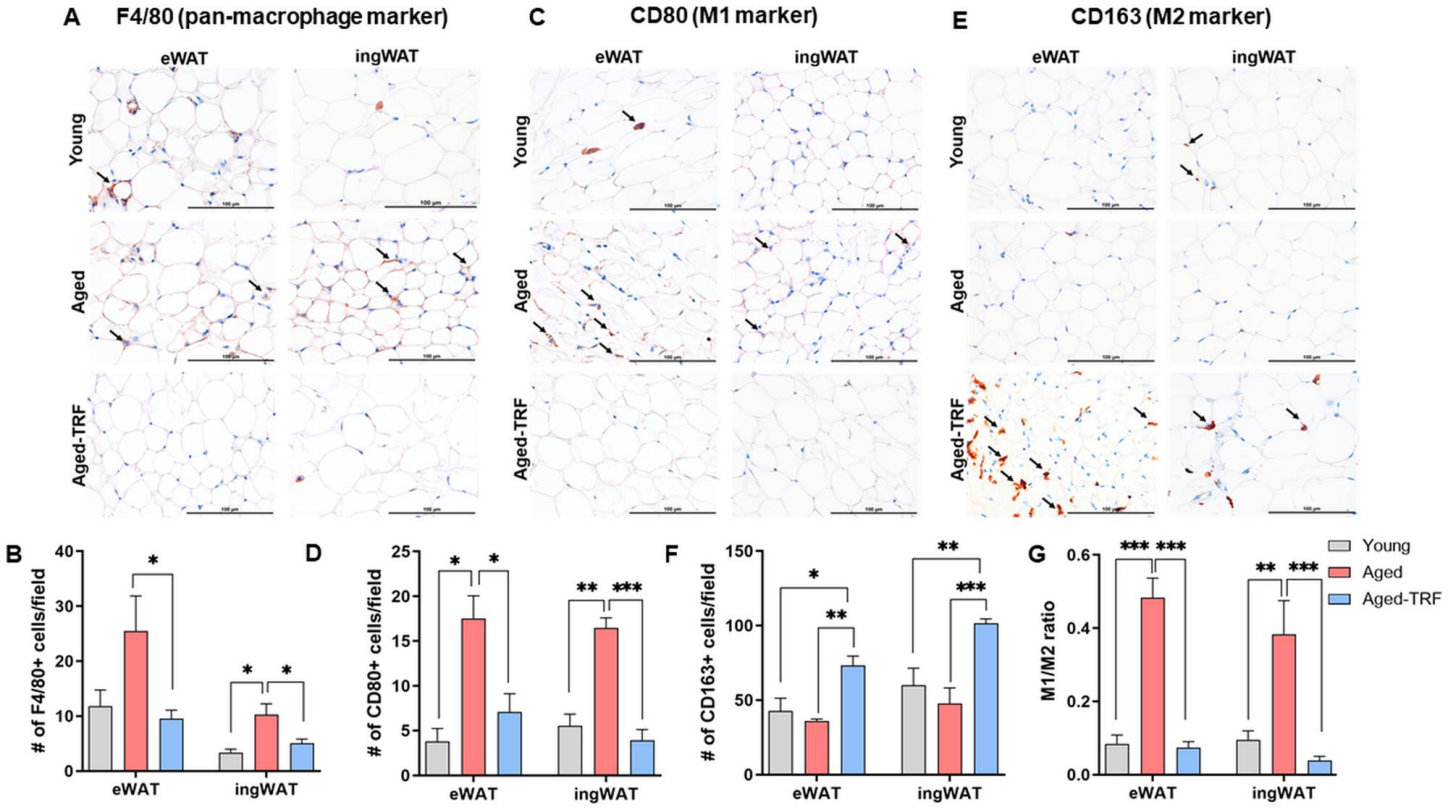
Age-related macrophage infiltration and unfavorable macrophage polarization are reversed by TRF. Representative IHC images of eWAT and ingWAT and their quantification for (A, B) F4/80 staining (pan-macrophage marker), (C, D) CD80 (M1 marker), and (E, F) CD163 (M2 marker), respectively. Black arrows indicate macrophages stained with brown color. Note that a higher number of F4/80+ and CD80+ macrophages were observed in the aged adipose tissue, while a higher number of CD163+ macrophages were noted in the aged-TRF animals. (G) M1/M2 ratio in WAT depots. *n* = 3-6/group. Data are presented as mean ± SEM, *n* = 3-6/group. *p < .05, **p < .01, and ***p < .005 between the indicated groups. eWAT, epididymal white adipose tissue; ingWAT, inguinal white adipose tissue; TRF, time-restricted feeding; WAT, white adipose tissue.

**Figure 7:**
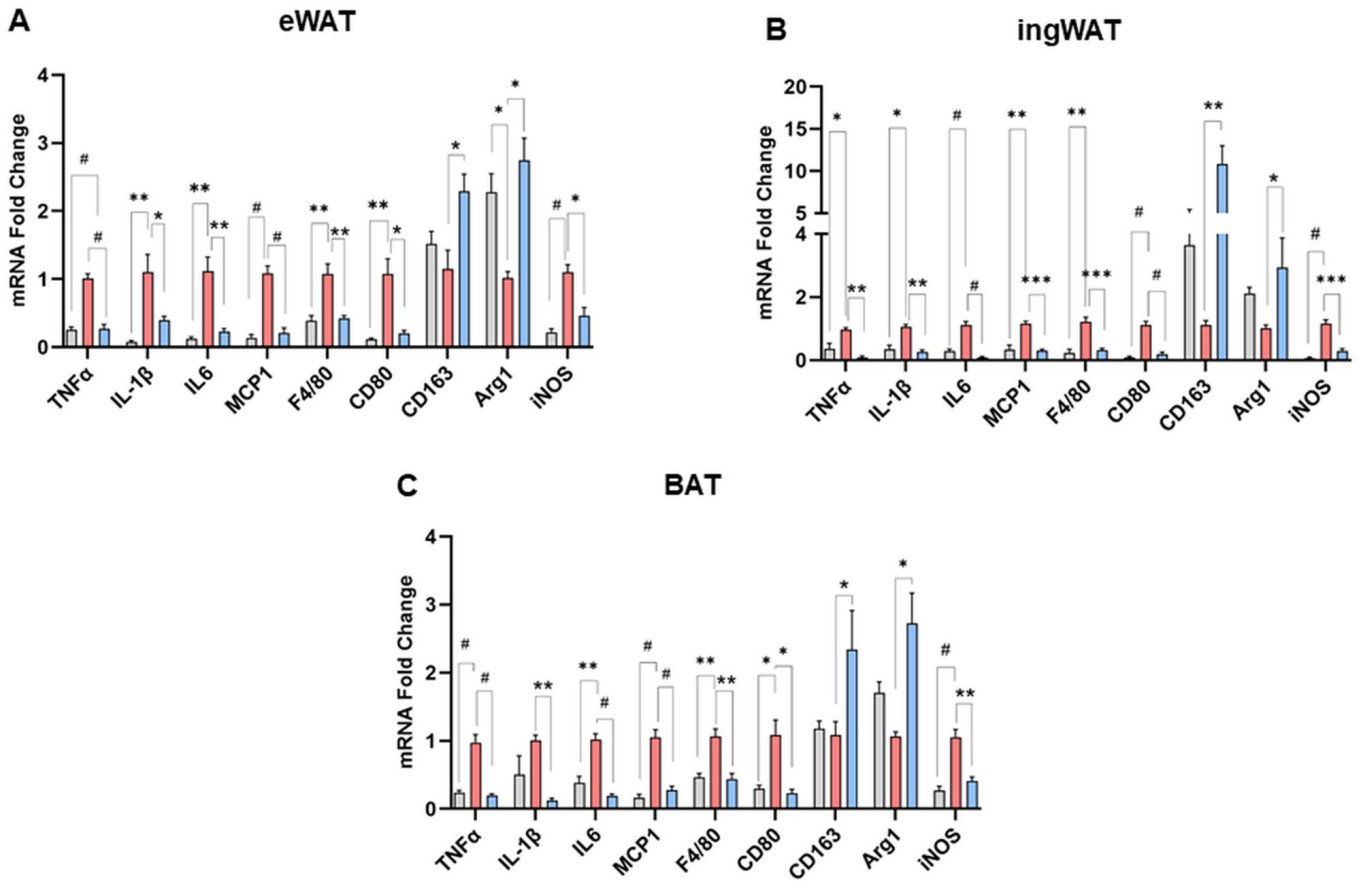
TRF Attenuated age-related increase in the expression of pro-inflammatory markers in adipose tissue. (A-C) Gene expression levels of pro-inflammatory and macrophage markers measured by real-time PCR in eWAT, ingWAT, and BAT, respectively. Data (mean ± SEM) were normalized against the housekeeping gene, GAPDH, in all the samples. *p < .05, **p < .01, ***p < .005, and ^#^*p* < .0001 between the indicated groups. BAT, brown adipose tissue; eWAT, epididymal white adipose tissue; GADPH, Glyceraldehyde-3-phosphate dehydrogenase; ingWAT, inguinal white adipose tissue; PCR, polymerase chain reaction; TRF, time-restricted feeding.

Aging is associated with increased recruitment and activation of pro-inflammatory M1 macrophages and loss of protective M2 macrophages in the WAT, which contributes to both local and systemic inflammation and insulin resistance.^28–30^ Hence, we investigated whether TRF impacted adipose macrophage recruitment and polarization in aged mice. To assess macrophage infiltration into WAT, we quantified the number of cells positive for F4/80, a pan-macrophage marker. TRF significantly reduced age-related accumulation of F4/80+ macrophages in both WAT depots (Figure 6A and B). We next evaluated macrophage polarization in WAT depots using CD80 as a marker for M1 macrophages and CD163 as a marker for M2 macrophages. In line with previous findings,^28^ aging increased the number of M1 macrophages in both eWAT and ingWAT compared to young controls (Figure 6C and D). Conversely, TRF significantly ablated age-related M1 macrophage accumulation in aged WAT depots (Figure 6C and D). In contrast to M1 macrophages, we did not observe a significant effect of aging on M2 macrophage numbers in WAT (Figure 6E and F). However, TRF significantly increased the number of M2 macrophages in both WAT depots when compared to both young and age-matched ad-libitum controls (Figure 6E and F). TRF also attenuated the age-related increase in the M1/M2 ratio in both WAT depots (Figure 6G), indicating a favorable shift in adipose tissue macrophage polarization toward an anti-inflammatory phenotype in the WAT of aged mice. Gene expression analysis revealed a similar pattern, with TRF upregulating M2 markers such as *Arg1* and *CD163* while downregulating M1 markers including *CD80* and *iNOS* in both WAT and BAT depots (Figure 7A-C). Additionally, TRF attenuated the age-related increase in the expression of multiple pro-inflammatory genes, including *TNFα*, *IL1β, IL6*, and *MCP1*, in both WAT and BAT depots (Figure 7A-C).

### TRF reverses age-related fibrosis in adipose tissue

**Figure 8:**
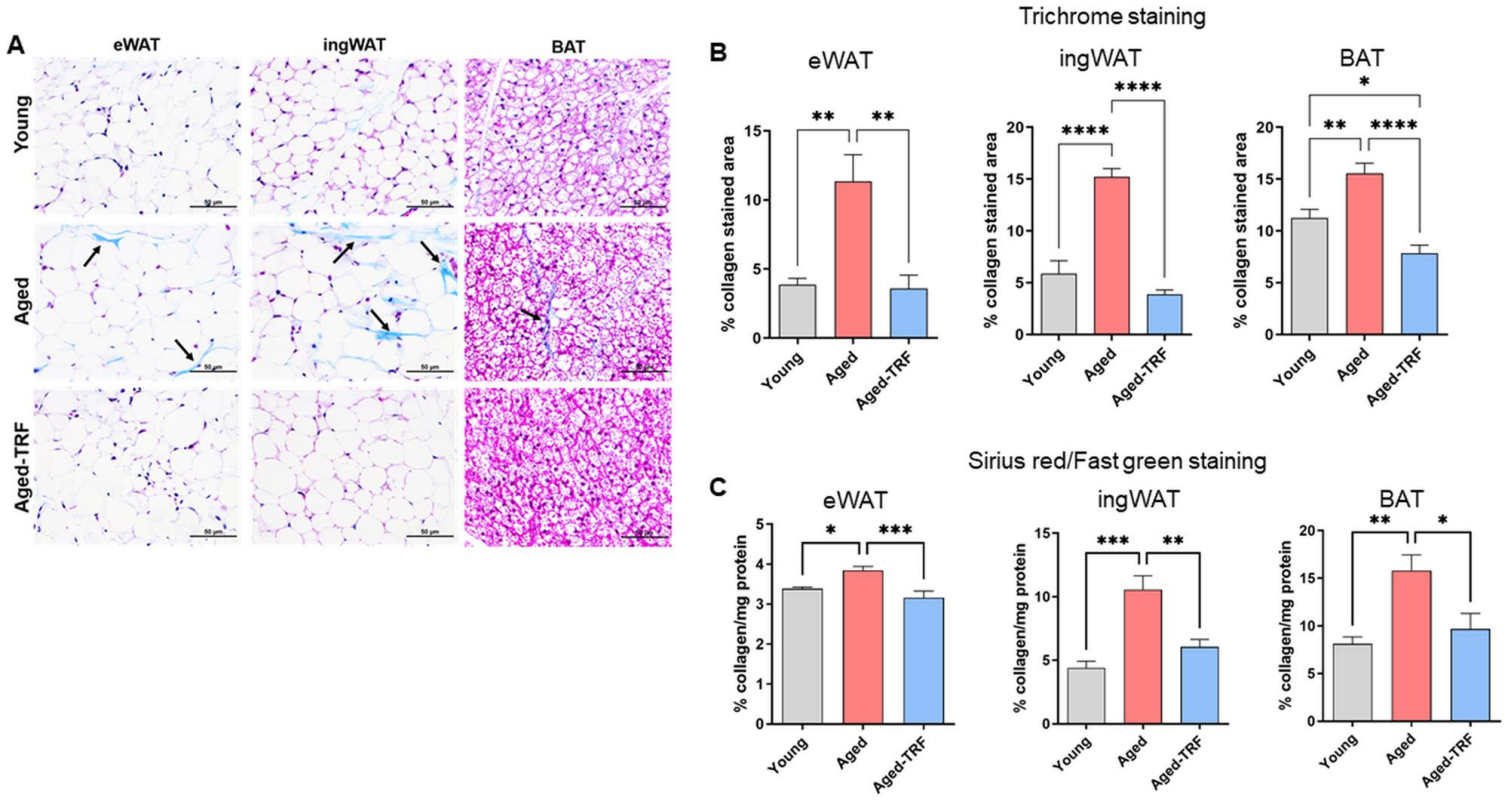
TRF reverses age-related fibrosis in adipose tissue. (A) Representative images of Masson’s Trichrome staining for collagen in adipose sections from young, aged, and aged-TRF animals. Black arrows point to blue-stained collagen fibers in adipose sections. (B) Image analysis from Trichrome staining showing % collagen-stained area in adipose sections. *n* = 5-6/group. (C) Semiquantitative analysis of collagen levels using Sirius Red/Fast Green staining after normalization to total protein levels. *p < .05, **p < .01, ***p < .005, and ****p < .001 between the indicated groups. BAT, brown adipose tissue; eWAT, epididymal white adipose tissue; ingWAT, inguinal white adipose tissue; TRF, time-restricted feeding.

Aging is also associated with increased fibrosis in WAT,^4^^,^^14^ which negatively affects adipose plasticity and promotes inflammation. We assessed adipose fibrosis using trichrome staining in fixed adipose sections. The collagen-stained areas (blue in Figure 8A) indicate the fibrotic areas in the adipose sections. In line with previous studies,^4^^,^^14^ we observed an age-related increase in collagen deposition in both WAT and BAT depots when compared to young controls (Figure 8B). TRF significantly decreased collagen-stained areas in all the adipose depots when compared to the age-matched ad-libitum controls (Figure 8B). We also performed a semiquantitative analysis of collagen levels using Sirius Red/Fast Green collagen staining kit. In line with the trichrome staining results, TRF attenuated the age-related increase in adipose collagen levels (Figure 8C), indicating that TRF mitigates age-related fibrosis in adipose tissue.

## Discussion

Intermittent fasting, including TRF and related strategies like alternate-day fasting, has been reported to confer metabolic benefits in younger populations;^24^^,^^31–33^ however, its impact on the aged population remains poorly understood. In the current study, we investigated the impact of chronic TRF on energy metabolism and adipose function, specifically focusing on beiging response, mitochondrial function, and inflammation in aging. Our findings demonstrate that chronic TRF promotes metabolic adaptation to fluctuations in energy availability and restores adipose tissue health during aging by reversing the age-related decline in the beiging response, reducing inflammation and fibrosis, and enhancing mitochondrial function. These findings are consistent with prior reports on the benefits of TRF on metabolism in younger animals^19^^,^^34^^,^^35^ and suggest that TRF is a promising nonpharmacological strategy to mitigate adipose dysfunction and improve metabolic health in aging.

Our results demonstrate that TRF induces metabolic adaptation in aged mice. Specifically, TRF results in elevated energy expenditure, oxygen consumption, and carbohydrate oxidation during the active feeding period, and suppressed metabolism during the fasting period. The observed increase in RER and preferential carbohydrate use during the dark phase further reflects enhanced metabolic flexibility and temporal coupling between nutrient intake and energy utilization. Together, this phenotype indicates that TRF reprograms energy metabolism in aged mice to align with nutrient availability, counteracting the loss of circadian coordination and metabolic adaptability typically observed with aging. One of the mechanisms that promotes increased energy expenditure is the activation of nonshivering thermogenesis in the adipose tissue. Consistent with this idea, TRF induced the formation of thermogenic beige adipocytes characterized by an increase in UCP1 expression and multilocular appearance within the WAT of aged mice. Other studies have also reported a similar phenomenon in response to intermittent fasting,^24^^,^^36^^,^^37^ and our results suggest that fasting-mediated WAT beiging is also preserved in aged mice. Interestingly, these metabolic changes in energy expenditure and adipose beiging occurred without significant changes in body weight or fat mass in aged mice. Age-related reductions in fat mass, specifically in the subcutaneous depot, have been associated with insulin resistance and increased frailty.^38^ Hence, it is likely that retention of fat mass during TRF might be an adaptive mechanism to improve adipose function while preserving systemic metabolic health in aging. Future studies with quantification of individual fat pads will address this question.

The mechanisms mediating TRF-induced beiging are multifaceted. First, TRF may decrease the senescence burden and increase the proliferation of adipose progenitor cells, thereby inducing the formation of UCP1+ adipocytes.^39^ TRF-induced activation of adipogenesis is also evident in the increased number of smaller adipocytes in our study. Second, fasting during TRF likely activates WAT lipolysis, resulting in the release of free fatty acids (FFA), which is a known activator and a fuel source for UCP1+ thermogenic adipocytes. TRF-induced adipose lipolysis is also highly relevant for its systemic effects, as FFAs are precursors for hepatic ketone body generation. Third, UCP1 expression is regulated by the core circadian clock,^40^ suggesting that feeding alignment with the active cycle is another potential mechanism for TRF-mediated adipose thermogenesis.^25^ Lastly, there is growing evidence that gut microbiota-derived metabolites can activate adipose browning,^41^ indicating that TRF-induced changes in the gut microbiome may also contribute to this phenomenon. Beige thermogenic adipocytes not only increase fuel utilization and energy expenditure but also secrete beneficial adipokines (adiponectin), bioactive peptides, and lipid molecules, which have implications for the anti-aging systemic effects of TRF.

Another important finding from the present study is that TRF restored the classical BAT phenotype in aging. TRF demonstrated increased UCP1 protein levels and the restoration of multilocular adipocyte appearance in BAT. In contrast to other studies that reported intermittent fasting-induced selective activation of beiging in WAT,^41^^,^^42^ our study demonstrates that TRF exerts broad thermogenic effects across multiple adipose depots, potentially enhancing energy expenditure and systemic energy metabolism. Chronic inflammation and fibrosis are interconnected features of the aging WAT arising from pathological activation of infiltrating macrophages.^43^ Results from the current study showed that TRF attenuated both age-related inflammation and fibrosis, likely mediated through the alternate activation of macrophages (increased M2: M1). Increased M2 macrophages in the aged WAT favor a shift toward an anti-inflammatory state, leading to reduced inflammation and secretion of pro-fibrotic factors in the adipose tissue. While we did not profile other adipose immune cells in this study, recent studies have reported that intermittent fasting restores eosinophil numbers in the gonadal WAT of aged mice.^39^ In addition to modulating inflammation, both M2 macrophages and eosinophils have been directly linked to adipose browning,^44^^,^^45^ and hence their role in TRF-mediated WAT beiging warrants further investigation. Given its role in immune regulation, adipose tissue has also been implicated in exacerbating outcomes of viral infections such as COVID-19, particularly in older and obese individuals, where increased ACE2 expression and chronic inflammation may promote viral persistence and severe disease.^46^

TRF-mediated rejuvenation of the adipose microenvironment also enhanced mitochondrial metabolism in the WAT and BAT depots. A recent study similarly reported improved mitochondrial fusion and increased ATP production in the inguinal WAT of obese mice subjected to an intermittent fasting regimen.^47^ While the precise molecular mechanisms underlying TRF-induced improvements in mitochondrial metabolism remain unclear, some studies suggest a role for SIRT3, a mitochondrial deacetylase, in regulating key enzymes involved in fatty acid oxidation, the TCA cycle, and ATP synthesis.^48^^,^^49^ Beyond adipose tissue, TRF has also been shown to enhance mitochondrial function and redox status in the aged aorta,^27^ indicating that mitochondrial adaptation may represent a common signature of TRF’s systemic effects during aging. Although we demonstrate robust molecular and histological improvements in aged adipose tissue, we did not evaluate physical function or frailty indices. Given the growing use of digital technologies to quantify such outcomes in older populations,^50^ incorporating these tools into future metabolic intervention studies may help bridge the gap between tissue-specific changes and clinically meaningful outcomes.

Our study also has some limitations. First, the data presented here were generated exclusively in male mice. Given the growing body of evidence showing sex-specific differences in metabolic, neurovascular, and neurodegenerative aging trajectories, including recent findings on sex-dependent genetic and connectivity biomarkers in Alzheimer’s disease,^51^ future studies will address potential sex-specific effects of TRF in aging by including both sexes. Second, although mitochondrial biogenesis is coupled with the development of beige adipocytes, it remains to be determined whether TRF enhances mitochondrial metabolism by increasing mitochondrial quantity, improving quality, or both. Additionally, while this study primarily focused on UCP1-dependent thermogenesis, emerging evidence suggests that WAT thermogenesis can also occur through UCP1-independent mechanisms, including futile cycling of metabolic substrates such as fatty acids, creatine, and calcium.^52–56^ Whether TRF also activates these alternate pathways to promote energy expenditure warrants further investigation. In addition, this study did not include a young TRF group, which would have enabled direct comparison of the magnitude of TRF-induced responses between young and aged animals. However, given that TRF-induced WAT beiging and metabolic improvements in young animals are well established,^24^^,^^25^^,^^42^^,^^57^ our study was specifically designed to assess whether these beneficial effects are preserved during aging. Finally, we only assessed whole-body fat mass but did not perform depot-specific fat pad weight measurements to determine changes in fat distribution between subcutaneous and visceral depots. Therefore, we cannot address whether TRF altered fat distribution in aged mice. Our future studies will address these limitations. Although our findings in aged mice reveal promising mechanisms by which TRF improves adipose health, future work should incorporate broader comparative and translational aging frameworks to validate these effects across models and species.^58^

## Supplementary Material

glag085_Supplementary_Data

## Data Availability

The data that support the findings of this study are available from the corresponding author upon reasonable request.
