# Supplementary figures and images for: Time-restricted feeding improves metabolic flexibility, promotes beiging, and mitigates fibro-inflammation in the adipose tissue of aged mice
Source: J Gerontol A Biol Sci Med Sci. 2026 Mar 30;81(5):glag085. doi: 10.1093/gerona/glag085 (PMC13120883; doi:10.1093/gerona/glag085)

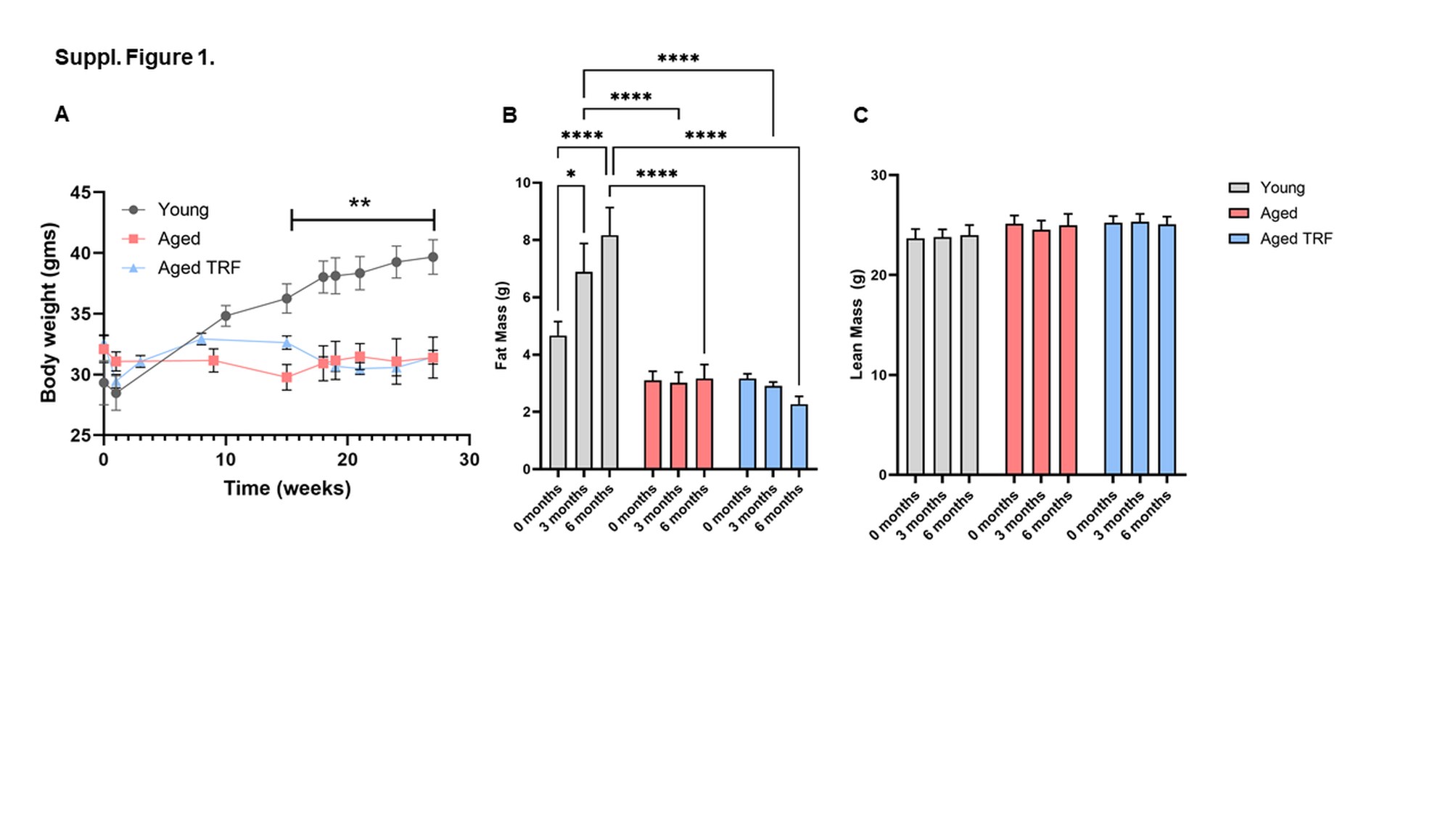

Supplement: glag085_Supplementary_Data [file glag085_supplementary_data.jpeg]
